# L Antigen Family Member 3 Serves as a Prognostic Biomarker for the Clinical Outcome and Immune Infiltration in Skin Cutaneous Melanoma

**DOI:** 10.1155/2021/6648182

**Published:** 2021-03-18

**Authors:** Jingjing Song, Cong Jin, Endong Chen, Xubin Dong, Libin Zhu

**Affiliations:** ^1^Department of Pediatric Surgery, The Second Affiliated Hospital & Yuying Children's Hospital of Wenzhou Medical University, Wenzhou, 325027 Zhejiang Province, China; ^2^Department of Children's Health Care, The Second Affiliated Hospital & Yuying Children's Hospital of Wenzhou Medical University, Wenzhou, 325027 Zhejiang Province, China; ^3^Department of Breast Surgery, The First Affiliated Hospital of Wenzhou Medical University, #1 Nan Bai Xiang Street, Zhejiang 325006, China

## Abstract

L Antigen Family Member 3 (LAGE3) is an important RNA modification-related protein. Whereas few studies have interrogated the LAGE3 protein, there is limited data on its role in tumors. Here, we analyzed and profiled the LAGE3 protein in skin cutaneous melanoma (CM) using TCGA, GTEx, or GEO databases. Our data showed an upregulation of LAGE3 in melanoma cell lines compared to normal skin cell lines. Besides, the Kaplan–Meier curves and Cox proportional hazard model revealed that LAGE3 was an independent survival indicator for CM, especially in metastatic CM. Moreover, LAGE3 was negatively associated with multiple immune cell infiltration levels in CM, especially CD8^+^ T cells in metastatic CM. Taken together, our study suggests that LAGE3 could be a potential prognostic biomarker and might be a potential target for the development of novel CM treatment strategies.

## 1. Introduction

Skin cutaneous melanoma (CM) is one of the most life-threatening types of skin cancer [1]. CM is characterized by quick lymph node or distant metastases and accounts for 72% of skin cancer mortality [2]. Due to the inefficiency of standard treatment options against melanoma, the use of immunotherapy has drastically improved the disease outcomes [3, 4]. Inhibitors of immune checkpoints such as cytotoxic T-lymphocyte antigen-4 (CTLA-4), programmed death receptor-1 (PD-1), and its associated ligand (PD-L1) have demonstrated remarkable clinical effect and prolong survival in a considerable number of patients [5–7]. Nevertheless, because the tumor microenvironment in CM is very complex, there is a need to investigate novel prognostic biomarkers and potential targets for immunotherapy in patients with CM.

The molecular characteristics of tumor-immune interaction are related to its diagnostic potential in melanoma. The development of melanoma is a dynamic process. Plenty of studies have proposed molecular characteristics based on gene expression for the prognosis of melanoma patients [8–10]. The L Antigen Family Member 3 (LAGE3) is ubiquitously expressed in the anticodon stem-loop of tRNAs decoding ANN codons [11]. The LAGE3 protein, a component of the tRNA threonylcarbamoyladenosine metabolic complex, plays a role in the RNA polymerase II-mediated positive transcription regulation [12] and affects translation accuracy and efficiency [13]. Previous studies have shown that LAGE3 is one of the most frequently upregulated RNA modification-related proteins in multiple cancer types [14]. Besides, a previous study demonstrated that a recessive mutation in the LAGE3 gene encodes one of four subunits in Galloway-Mowat syndrome (GAMOS) [15]. Besides, the immune-related prognostic analysis of LAGE3 in colorectal cancer, clear cell renal cell cancer, and malignant pleural mesothelioma has been documented [16–18]. It is important to identify a potentially reliable immune signature for melanoma metastasis. However, data on the clinical value and distinct function of the LAGE3 protein in CM remains scant.

In this study, we used bioinformatics tools, multiple CM cell lines, and normal human epidermal melanocytes to explore the expression of LAGE3 and its prognostic value in CM development. We exploited Gene Ontology (GO), Kyoto Encyclopedia of Genes and Genomes (KEGG), and Gene Set Enrichment Analysis (GSEA) to dissect the functional roles of LAGE3 in tumor-infiltrating immune cells (TIICs) in the tumor microenvironment (TME). Taken together, we provide evidence that increased LAGE3 could be a novel prognostic biomarker for worse outcomes in CM patients, and its correlation with immune cells might define the prognostic mechanism of CM.

## 2. Materials and Methods

### 2.1. Datasets

The transcriptome RNA-seq profiles and corresponding clinical information of TCGA-SKCM samples were downloaded from Genomic Data Commons (https://portal.gdc.cancer.gov/) [19]. We obtained 556 RNA-seq profiles of normal skin tissues from the Genotype-Tissue Expression (GTEx) dataset [20] and one matched normal sample from TCGA-CM cohort to enlarge the sample size. Among TCGA-CM, we split the melanoma profiles into primary and metastatic subtypes based on official annotated information. Batch effects were removed using the “normalizeBetweenArrays” function in the “limma” package when we combined transcriptomic data from TCGA and GTEx datasets. RNA-seq and sample profiles used for the evaluation of LAGE3 expression (GSE3189 [21], GSE98394 [22], and GSE46517 [23]) and prognostic value (GSE19234 [24], GSE98394, GSE65904 [25], and GSE22153 [26]) were obtained from the Gene Expression Omnibus (GEO) database. In summary, we gathered six publicly available datasets for our analyses (Table 1). For TCGA, GTEx, and GEO cohorts, the expression profiles were transformed into log2(TPM + 1) for downstream analyses.

### 2.2. Cell Culture

Normal human epidermal melanocytes, adult, the lightly pigmented donor (HEMa-LP) were obtained from Invitrogen and cultured in Medium 254 (Cascade Biologics), supplemented with human melanocyte growth supplement (Cascade Biologics). Melanoma cell lines A375, M21, and MELRM were purchased from the Cell Bank of the Chinese Academy of Sciences (Shanghai, China). On the other hand, the melanoma cell line (SK-MEL-2) was purchased from the American Type Culture Collection (ATCC). All melanoma cell lines were maintained in DMEM (Gibco, USA) containing 10% FBS (Gibco, USA) and 100 U/ml of penicillin/streptomycin (Invitrogen, USA). We cultured the cells in a humidified incubator with a 5% CO_2_ atmosphere at 37°C.

### 2.3. RNA Extraction and qRT-PCR

Total RNA was extracted from the cell lines using the TRIzol reagent (Thermo Fisher Scientific, USA) following the manufacturer's instructions. All the RNA samples were temporarily stored at -80°C. The isolated RNA samples were measured at 260/280 nm to ensure the quality and concentration of RNA. The qRT-PCR reaction was performed using the ReverTra Ace qPCR RT Kit (Toyobo, Japan) following the manufacturer's protocol. The relative expression of the LAGE3 mRNA was calculated using the 2^-*ΔΔ*CT^ method with GAPDH as an endogenous control. The primer sequences used are as follows: LAGE3 forward primer, 5′-GGATCTCACAGTGAGTGGCAGG-3′; LAGE3 reverse primer, 5′-GAAAGCTGGTCAAGAAAGTTGATG-3′; GAPDH forward primer, 5′-GTCTCCTCTGACTTCAACAGCG-3′; and GAPDH reverse primer, 5′-ACCACCCTGTTGCTGTAGCCAA-3′.

### 2.4. Western Blot

The RIPA buffer (Beyotime Biotechnology, Shanghai, China) supplemented with protease inhibitor cocktail (Beyotime Biotechnology, Shanghai, China) and PMSF (Beyotime Biotechnology, Shanghai, China) was used to lyse the cells. The lysates were centrifuged at 12,000 × g. Total protein concentration was quantified using BCA analysis. The protein in the lysate was then separated using sodium dodecyl sulfate-polyacrylamide gel electrophoresis (SDS-PAGE) and subsequently electrotransferred to a PVDF membrane (Millipore, Bedford, MA). The PVDF membrane was blocked with 5% skim milk (BD Biosciences, USA). The target proteins were detected by incubating the membrane at 4°C overnight with the primary anti-LAGE3 antibody (#PA5-46520, Invitrogen) (1 : 1000) and primary anti-GAPDH antibody (1 : 5000, #AP0063, Bioworld Technology). Next, a secondary antibody goat anti-rabbit IgG (1 : 5000, #BL003A, Biosharp) was added to the membrane and incubated for two hours at room temperature. Finally, the protein bands were visualized by the ECL detection kit (Beyotime Biotechnology, Shanghai, China). The bands were scanned and photographed by the ChemiDoc MP image system (Bio-Rad, USA) and quantitated by the Image Lab software.

### 2.5. Gene Expression Analysis

GSE3189, GSE98394, GSE46517, or TCGA-CM had 45, 51, 104, or 471 melanoma patients, respectively. The CM and normal skin tissue immunohistochemistry (IHC) images from the Human Protein Atlas (HPA) [27] were used for the identification of subcellular localization and evaluation of the LAGE3 protein expression. Immunohistochemical staining was performed using an anti-LAGE3 antibody (Cat. No. HPA036122).

### 2.6. Survival Analysis

The overall survival (OS), disease-specific survival (DSS), and progression-free interval (PFI) outcome of the CM samples were obtained from TCGA-Clinical Data Resource (CDR) [28]. Kaplan–Meier curves with the log-rank test were performed by the “survival” package. The clinicopathological factors, along with the expression of the LAGE3 protein, were used for univariate and multivariate Cox regression analyses by the “survival” package. Only significant factors with a *p* value < 0.05 in the univariate analyses were included in multivariable analyses. The median expression of the LAGE3 protein was used to divide it into either LAGE3^low^ or LAGE3^high^ groups.

### 2.7. Functional Enrichment Analysis

We used the “cor.test” function in R with the Spearman method to evaluate the correlation analysis. Highly ranked coexpressed genes with LAGE3 were selected (Spearman correlation value > 0.4 or <−0.4, *p* < 0.001). We then performed Gene Ontology (GO) and Kyoto Encyclopedia of Genes and Genomes (KEGG) analyses by “clusterProfiler” package, while “ggplot2” and “enrichplot” packages were used for visualization. We performed the Gene Set Enrichment Analysis (GSEA) using the GSEA-4.0.3 software [29, 30]. GSEA hallmark and C7 gene set v6.2 collections were downloaded from the Molecular Signatures Database as the target sets. The RNA-seq profiles of all TCGA-CM samples were used for GSEA, and only gene sets with an FDR *q* < 0.05 were considered significant.

### 2.8. Immune-Related Analysis

We estimated the proportion of immune and cancer cells (EPIC) as an efficient algorithm to simultaneously assess the fraction of cancer and immune cell types from bulk tumor gene expression data [31]. Tumor Immune Estimation Resource (TIMER) is a database that comprehensively characterizes the molecular tumor-immune interactions [32]. We utilized the EPIC and TIMER algorithms to explore the abundance of different cell types in the TME. For specific cell type survival analyses, TIMER was used to generate estimates for the clinical outcomes of various immune cell types in the TME. Quantile normalization was disabled using the RNA-seq TPM data. A set of marker genes for immune-related functions was obtained from Bindea et al. [33]. We used the Gene Set Variation Analysis R package to analyze the activity of immune-related functions based on TCGA-CM expression profile.

### 2.9. Statistical Analysis

We used the Mann–Whitney test or Wilcoxon signed-rank test for two-group analysis. Kruskal–Wallis one-way analysis of variance (ANOVA) was used for comparisons between the different groups. Survival analysis, cancer immunity-related analysis, and functional enrichment analysis were conducted in R version 4.0.0 and GraphPad Prism 8.1.0.

## 3. Results

### 3.1. Overview of LAGE3 Expression

**Figure 1:**
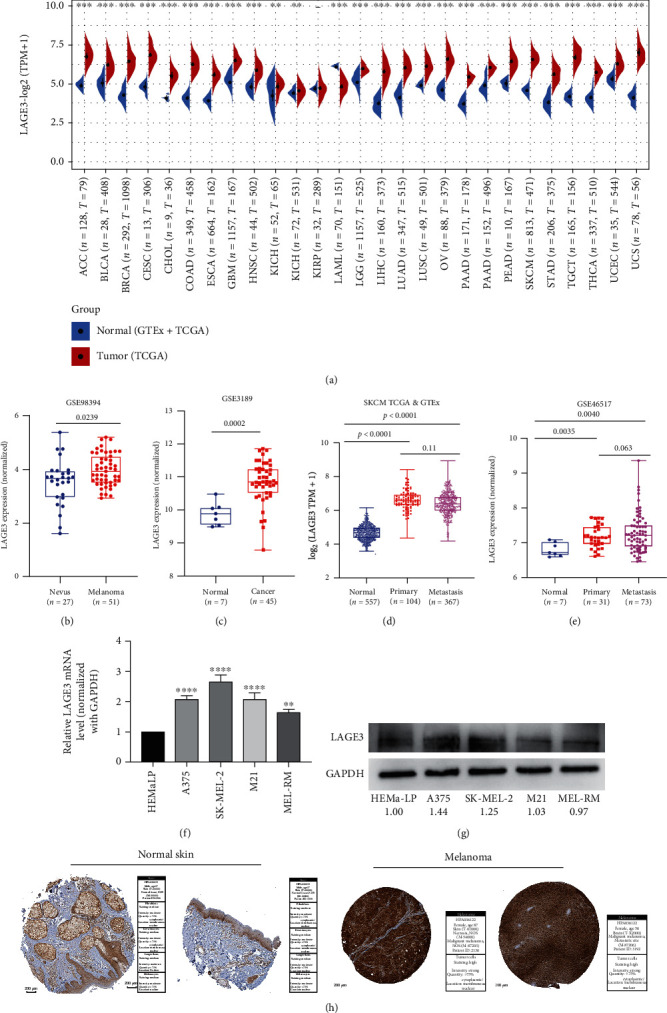
Expression analysis of LAGE3. (a) The mRNA expression of LAGE3 between the tumor and normal tissues (ACC, BLCA, BRCA, CESC, CHOL, COAD, ESCA, GBM, HNSC, KICH, KIRC, KIRP, LAML, LGG, LIHC, LUAD, LUSC, OV, PAAD, PRAD, READ, SKCM, STAD, TGCT, THCA, UCEC, and UCS) was assessed using tissues from TCGA and GTEx. (b–e) LAGE3 mRNA expression levels by (b) GSE98394, (c) GSE3189, (d) TCGA combined with GTEx, and (e) GSE46517. ANOVA was used for comparisons between the different groups. (f, g) LAGE3 mRNA expression levels and protein expression levels in normal human epidermal melanocytes (HEMa-LP) and melanoma cell lines (A375, SK-MEL-2, M21, and MEL-RM). The numbers were the expression of LAGE3 protein relative to GAPDH, and the normal epidermal cell expression was set as 1. (h) Representative immunohistochemistry images and detailed information about LAGE3 in melanoma tissues and normal tissues using HPA. ACC: adrenocortical carcinoma; BLCA: bladder urothelial carcinoma; BRCA: breast invasive carcinoma; CESC: cervical squamous cell carcinoma and endocervical adenocarcinoma; CHOL: cholangiocarcinoma; COAD: colon adenocarcinoma; ESCA: esophageal carcinoma; GBM: glioblastoma multiforme; HNSC: head and neck squamous cell carcinoma; KICH: kidney chromophobe; KIRC: kidney renal clear cell carcinoma; KIRP: kidney renal papillary cell carcinoma; LAML: acute myeloid leukemia; LGG: brain lower grade glioma; LIHC: liver hepatocellular carcinoma; LUAD: lung adenocarcinoma; LUSC: lung squamous cell carcinoma; OV: ovarian serous cystadenocarcinoma; PAAD: pancreatic adenocarcinoma; PRAD: prostate adenocarcinoma; READ: rectum adenocarcinoma; SKCM: skin cutaneous melanoma; STAD: stomach adenocarcinoma; TGCT: testicular germ cell tumors; THCA: thyroid carcinoma; UCEC: uterine corpus endometrial carcinoma; UCS: uterine carcinosarcoma.

Firstly, we compared the mRNA expression of LAGE3 between 27 kinds of tumors and normal tissues using TCGA and GTEx data (Figure 1(a)). In most cancer types, LAGE3 mRNA was significantly upregulated in the cancerous tissues compared with the normal tissues. To determine the LAGE3 mRNA expression in CM, we used the GSE3189, GSE98394, GSE46517, TCGA, and GTEx RNA-seq data to compare the LAGE3 gene expression between the cancerous and normal tissues. Data showed that LAGE3 mRNA was significantly upregulated in the CM compared with the normal tissues or nevus (Figures 1(b)–1(e)). Besides, the upregulation of the LAGE3 protein was observed in both the primary and metastatic CM samples (Figures 1(b)–1(e)), and there were no differences in the LAGE3 expression between the primary and metastatic CM groups (Figures 1(d) and 1(e)).

We then examined the LAGE3 expression pattern in the tumor and normal tissues. We explored the level of LAGE3 in normal human epidermal melanocytes (HEMa-LP) and melanoma cell lines (A375, SK-MEL-2, M21, and MEL-RM) by qRT-PCR and western blot. The results of qRT-PCR showed higher mRNA expression of LAGE3 in melanoma cell lines than in normal tissues. Similarly, the data demonstrated an upregulation of the LAGE3 protein in most melanoma cells compared to HEMa-LP cells (Figure 1(g)). Moreover, IHC staining pulled from the Human Protein Atlas showed high LAGE3 protein expressions in the cancerous tissues than in the normal tissues (Figure 1(h)). Thus, LAGE3 is upregulated in melanoma cells and tissues.

### 3.2. Identification of the Prognostic Value of LAGE3

**Figure 2:**
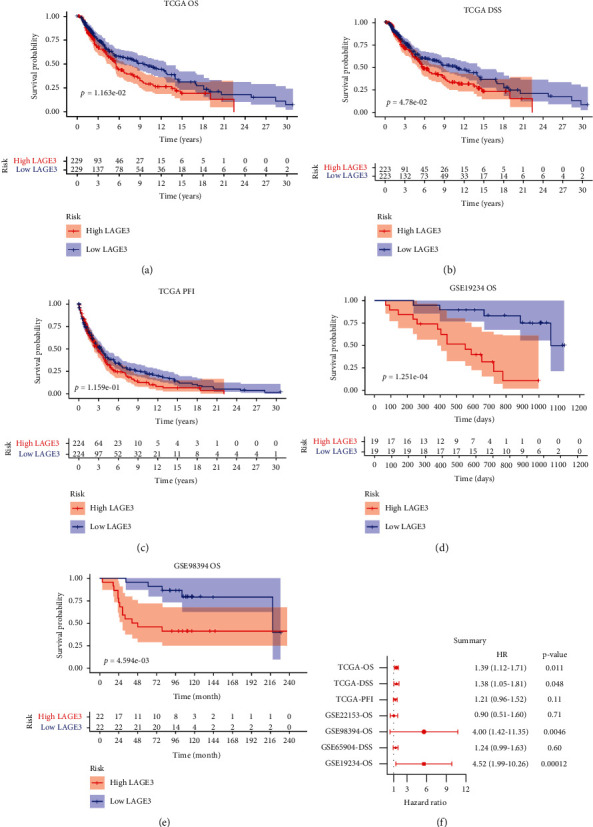
Kaplan–Meier plotter (log-rank test) and Cox regression analysis of the prognostic significance of LAGE3 in CM patients. (a–c) OS, DSS, and PFI survival curves between the LAGE3^low^ and LAGE3^high^ groups based on TCGA cohort. (d, e) OS curves between the LAGE3^low^ and LAGE3^high^ groups in the GSE19234 and GSE98394 cohorts. (f) Summary of multivariate Cox regression analysis of LAGE3 in different cohorts. OS: overall survival; DSS: disease-specific survival; PFI: progression-free interval.

To explore the potential of using LAGE3 as a prognostic biomarker in CM, we performed the Kaplan–Meier plotter based on TCGA database and several GEO public databases. Compared with the LAGE3^high^ group, our data showed that the LAGE3^low^ group was significantly associated with better OS (*p* = 0.012, Figure 2(a)) and DSS (*p* = 0.048, Figure 2(b)) in TCGA cohort. As shown in Figure 2(c), high LAGE3 levels were associated with poor PFI in CM patients (*p* = 0.12). The latter results from the GSE19234 and GSE98394 cohorts verified the significant association between LAGE3^high^ and shorter OS (Figures 2(d) and 2(e)).

In addition, Cox regression analyses were performed to confirm the prognostic significance of the LAGE3 protein. We then built the Cox regression models after adjusting for the impacts of known risk factors. Our analysis showed that patients with high levels of LAGE3 were associated with worse TCGA OS (hazard ratio (HR) = 1.39, 95% CI 1.12–1.71, and *p* = 0.011), TCGA DSS (HR = 1.38, 95% CI 1.05–1.81, and *p* = 0.048), GSE98394 OS (HR = 4.00, 95% CI 1.42–11.35, and *p* = 0.0046), and GSE19234 OS (HR = 4.52, 95% CI 1.99–10.26, and *p* = 0.00012, Figure 2(f)).

**Figure 3:**
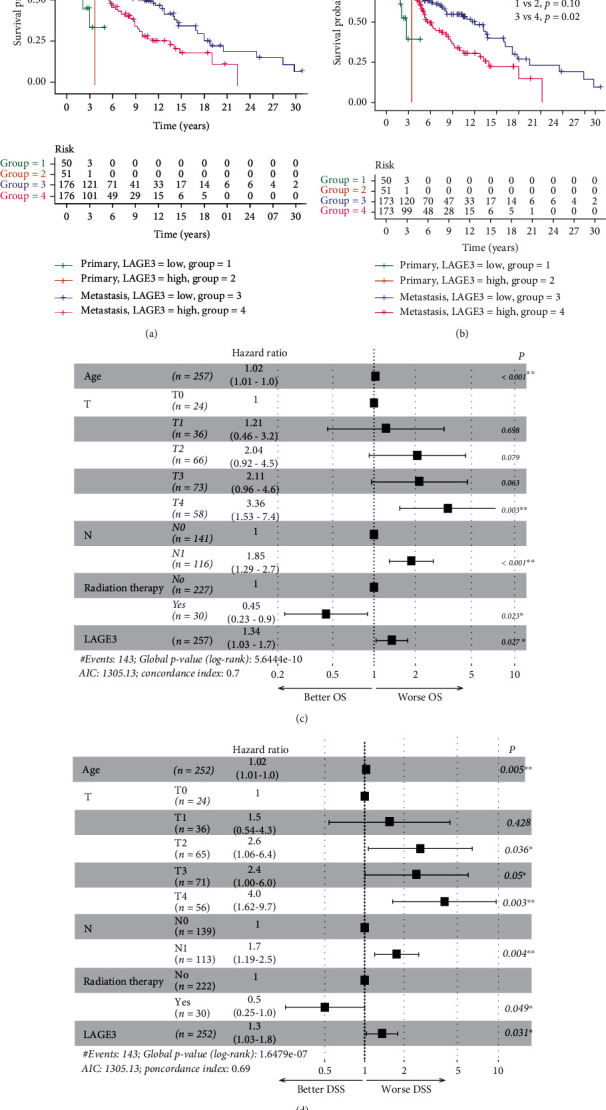
Prognostic significance of LAGE3 in metastatic CM. (a) OS and (b) DSS curves by primary and metastatic TCGA-CM samples between the LAGE3^low^ and LAGE3^high^ groups. (c, d) Multivariate Cox regression analysis of OS and DSS in metastatic TCGA-CM samples.

Although we examined the prognostic role of the LAGE3 in CM, the majority of the samples were collected from metastatic patients. We then analyzed survival outcomes in the primary and metastatic CM with LAGE3 expression. Our data showed that the LAGE3^high^ group was associated with lower survival rates in metastatic CM but not in primary CM (Figure 3(a)). Whereas there was no significant association between LAGE3 expression and DSS in primary CM, high LAGE3 expression was associated with worse DSS in metastatic CM (Figure 3(b)). The univariate Cox regression analysis of the metastatic TCGA-CM cohorts is shown in Supplementary Tables 1 and 2, and significant factors with a *p* value < 0.05 were included for further multivariate analyses. In the multivariable Cox regression analysis, age, tumor size (T), regional lymph nodes (N), radiation therapy, and LAGE3 expression were statistically significant prognostic factors for OS and DSS in the metastatic CM (Figures 3(c) and 3(d)). Taken together, these data suggested that high LAGE3 levels could lead to worse prognosis in CM patients, and LAGE3 expression might be a significant independent prognostic factor in patients with metastatic CM.

### 3.3. Functional Analysis and Predicted LAGE3 Signaling Pathways

**Figure 4:**
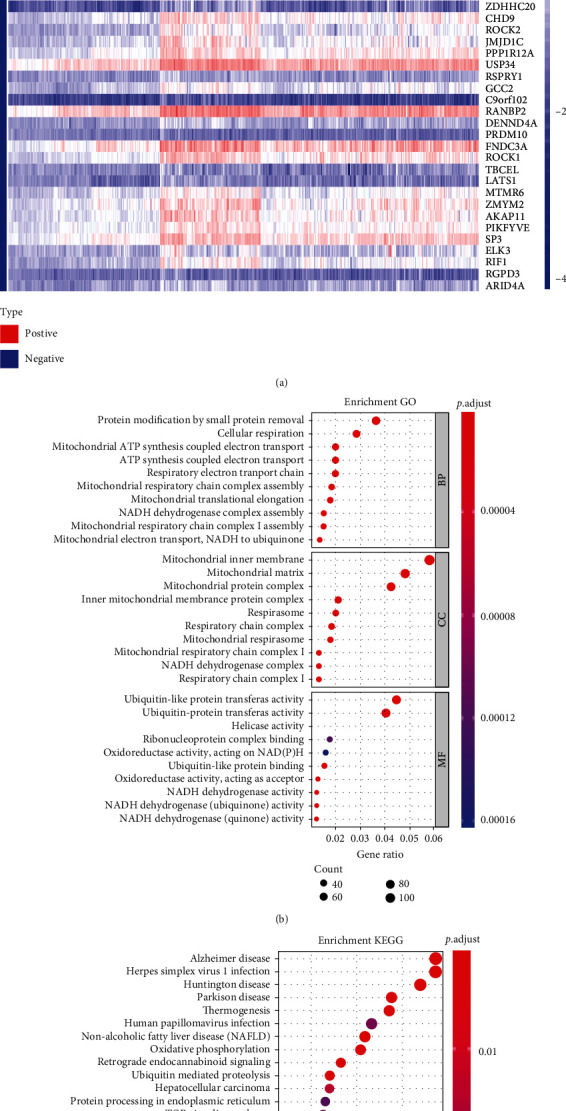
Coexpressed genes and functional enrichment analysis of the LAGE3. (a) Upregulated and downregulated coexpressed genes significantly associated with LAGE3 expression (FDR < 0.001 and Cor > 0.4 or Cor < −0.4). (b) The top 10 enrichment GO results of the BP, CC, and MF categories. (c) The top 30 enrichment results of the KEGG signal pathway.

To investigate the biological role of LAGE3 in CM, we profiled gene expression associated with LAGE3 based on TCGA-CM data. We used a gene expression matrix to construct the coexpression heat maps and uncover the LAGE3 mechanisms. Upregulated and downregulated genes significantly associated with LAGE3 expression were filtered (Figure 4(a), Supplementary Table 3). GO analysis clustered and established by bubble plots indicated that the biological processes (BP), such as protein modification by small protein removal, cellular respiration, mitochondrial ATP synthesis coupled electron transport, ATP synthesis coupled electron transport, or respiratory electron transport chain, were consistent with enrichment in respective cellular components (CC) and proposed molecular functions (MF) (Figure 4(b), Supplementary Table 4). Our data showed that the top 30 KEGG pathway terms were mostly involved in neurodegenerative diseases (Alzheimer's disease, Huntington's disease, and Parkinson's disease) and cancer-related terms (hepatocellular carcinoma, chronic myeloid leukemia, colorectal cancer, and renal cell carcinoma) (Figure 4(c), Supplementary Table 5).

We then implemented the GSEA to identify the potential signaling pathways. To explore more precise results, we explored each hallmark which conveyed a specific biological state or process and displayed coherent expression [34]. The overexpressed gene sets upregulated in the higher LAGE3 level subset were robustly enriched in adipogenesis, DNA repair, glycolysis, MYC targets, oxidative phosphorylation, UV response up, or xenobiotic metabolism. In addition, the negatively enriched genes in the LAGE3-deficient subset included KRAS signaling up, TGF-*β* signaling, and UV response down (Supplementary Figure 1a). Furthermore, using the GSEA, we found a significant correlation between numerous KEGG pathways and LAGE3. The pathways included Alzheimer's disease, lysosome, metabolism of xenobiotics by cytochrome P450, oxidative phosphorylation, Parkinson's disease, purine metabolism, pyrimidine metabolism, ribosome, TGF-*β* signaling pathway, and WNT signaling pathway (Supplementary Figure 1b). These results confirmed the KEGG pathway data (Figure 4(c)). Immunologic signatures collection (ImmuneSigDB) is composed of a set of genes representing cell types, states, and perturbations in the immune system. The ImmuneSigDB helps improve the biological understanding of immune processes [35]. Unlike in the low-LAGE3 expression group, the C7 collection defined by ImmuneSigDB showed enrichment of multiple immune functional gene sets in the high-LAGE3 expression group (Supplementary Figure 1c). These results give insight and systemic biological information on the LAGE3 protein in CM.

### 3.4. The Landscape of TIICs in CM

**Figure 5:**
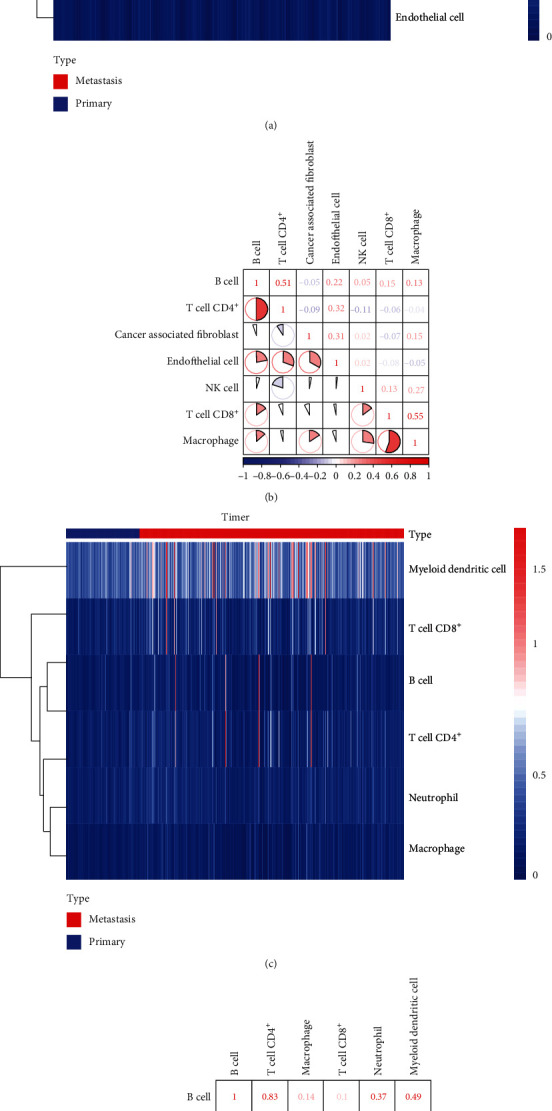
Correlation between TIICs in CM based on EPIC and TIMER. (a) The heat map shows the immune infiltration levels of the TIICs from the primary and metastatic TCGA-CM samples by EPIC. (b) The proportions of different TIIC subpopulations had a weak to medium correlation in tumor tissues by EPIC. (c) The heat map shows the immune infiltration levels of the TIICs from the primary and metastatic TCGA-CM samples by TIMER. (d) The proportions of different TIIC subpopulations had a weak to medium correlation in tumor tissues by TIMER. The redder color indicates a higher correlation, and the bluer color indicates a lower correlation.

TIICs are an integral component of the TME and correlate with tumor therapy response and prognosis [36]. We used two different algorithms to demonstrate the significant differences among the TIIC levels in the primary and metastatic CM. Heat map data suggested that TIICs like cancer-associated fibroblasts, B cells, CD8^+^ T cells, macrophages, and CD4^+^ T cells were highly expressed in metastatic CM compared to primary CM by EPIC (Figure 5(a)). Besides, EPIC demonstrated that different subpopulations of the TIICs in tumor tissues had a weak to moderate correlation (Figure 5(b)). Similarly, TIMER also showed high expression of CD4^+^ T cells, B cells, CD8^+^ T cells, and macrophages in metastatic CM than in primary CM. A similar trend was found in myeloid dendritic cells (Figure 5(c)). Different subpopulations of TIICs in tumor tissue had a weak to high correlation in TCGA cohort (Figure 5(d)).

### 3.5. Correlation between LAGE3 and Immune Cell Infiltration Levels in CM

**Figure 6:**
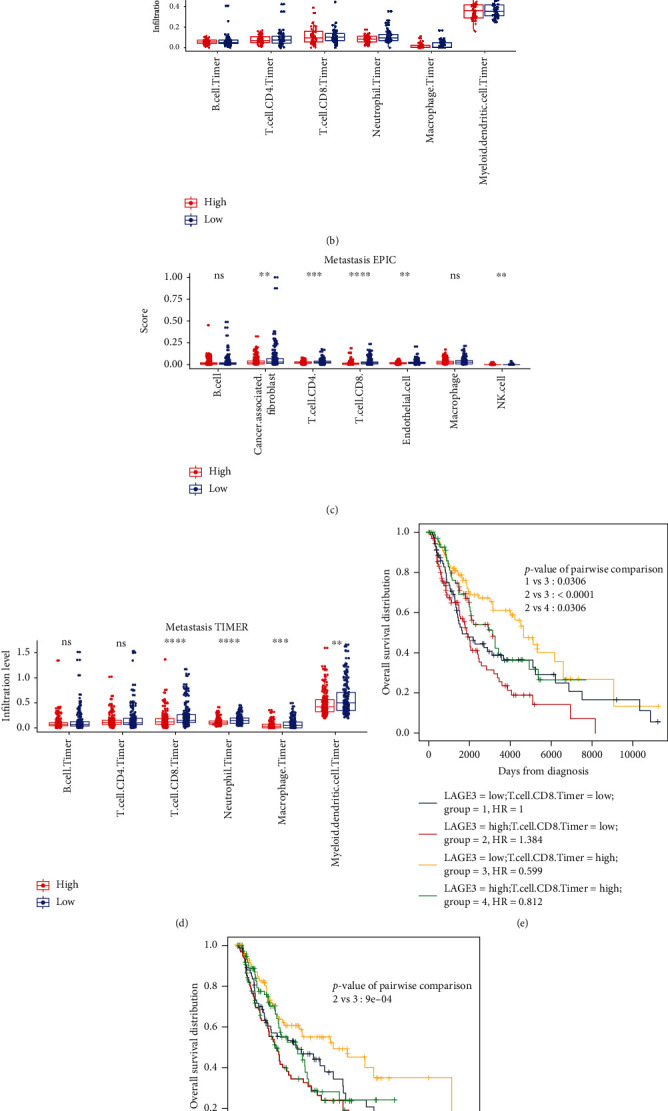
Correlation between LAGE3 and TIICs in primary and metastatic CM. (a, b) The immune infiltration levels of the TIICs from the primary CM samples by EPIC and TIMER. The Mann–Whitney test was used in group analysis. (c, d) The immune infiltration levels of the TIICs from the metastatic CM samples by EPIC and TIMER. The Mann–Whitney test was used in group analysis. (e, f) Survival analysis for the high/low LAGE3-expression groups (metastatic melanoma) and high/low infiltration of CD8 T cell groups by EPIC and TIMER.

Here, we combined the EPIC and TIMER algorithms to investigate the immune infiltration level in the primary and metastatic CM patients. In the primary CM, the EPIC algorithm showed that there was a high expression of CD4^+^ T cells and CD8^+^ T cells in the LAGE3^low^ group compared to the LAGE3^high^ group (Figure 6(a)). On the other hand, TIMER only showed that neutrophils were differentially expressed between the LAGE3^low^ and LAGE3^high^ groups in the primary CM (Figure 6(b)). Comparative EPIC data from the metastatic CM showed that cancer-associated fibroblasts, CD4^+^ T cells, CD8^+^ T cells, endothelial cells, and natural killer (NK) cells had higher immune infiltration levels in the LAGE3^low^ group than in the LAGE3^high^ group (Figure 6(c)). Moreover, using TIMER, low LAGE3 expression was correlated with gene signatures featuring immune activation, such as overexpression of the CD8^+^ T cells, neutrophils, macrophages, or myeloid dendritic cells in metastatic CM (Figure 6(d)). Then, we used EPIC and TIMER algorithms to perform survival analysis for the high/low LAGE3-expression groups (metastatic melanoma) and high/low infiltration of CD8 T cell groups. The result showed that in both the LAGE3^low^ and LAGE3^high^ groups, high immune infiltration of CD8 T cells was significantly associated with better OS by EPIC (Figure 6(e)). This trend was also identified by the TIMER algorithm in Figure 6(f). Therefore, LAGE3 might play a vital role in the immune cell infiltration in CM, especially in metastatic CM.

### 3.6. Prognostic Value of the Immune Cells in CM

**Figure 7:**
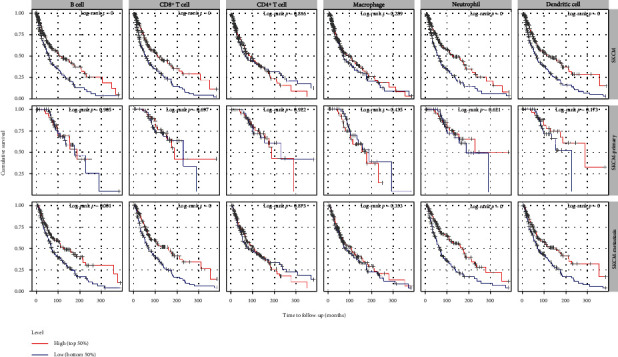
Correlation between expression levels plotted above or below the median of B cells, CD8^+^ T cells, CD4^+^ T cells, macrophages, neutrophils, and dendritic cells and primary, metastatic, and all CM patient cumulative survival.

Through the TIMER database, we found a significant correlation between the high levels of B cells, CD8^+^ T cells, neutrophils, and dendritic cells and better CM outcome, especially in metastatic CM (Figure 7). Taken together, the data inferred that immune activation and longer clinical survival in the LAGE3^low^ group may contribute to the robust predictive value to immune therapeutic sensitivity.

### 3.7. Correlation between LAGE3 and Immune-Related Functions in CM

**Figure 8:**
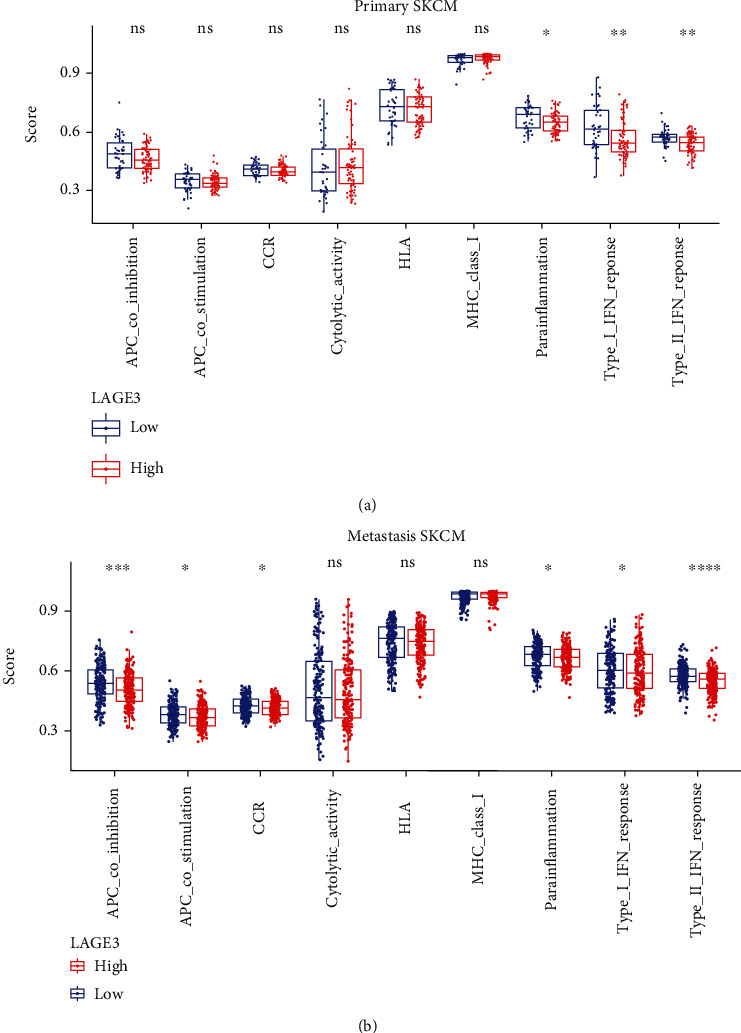
Correlation between LAGE3 and immune-related functions in CM. (a, b) Immune gene sets show lower enrichment levels in the LAGE3^high^ group than in the LAGE3^low^ group in (a) primary and (b) metastatic CM. The Mann–Whitney test was used in group analysis.

We used the Gene Set Variation Analysis to compare the immune-related genes in the LAGE3^high^ and LAGE3^low^ groups. The data showed that there were significant differences in the components of immune infiltration between the two groups in the primary and metastatic CM (Figures 8(a) and 8(b)). In the primary CM, the proportions of parainflammation, type I IFN response, and type II IFN response were significantly lower in the LAGE3^high^ group than in the low-risk group (*p* < 0.05) (Figure 8(a)). On the other hand, in the metastatic CM, the proportions of APC coinhibition, APC costimulation, cytokine and cytokine receptor (CCR), parainflammation, type I IFN response, and type II IFN response were significantly lower in the LAGE3^high^ group than in the low-risk group (*p* < 0.05) (Figure 8(b)). Thus, LAGE3 might suppress the immune-related functions, especially in metastatic CM.

## 4. Discussion

LAGE3 is an important component of the universal tRNA-modifying EKC/KEOPS complex [37, 38]. However, the functions of LAGE3 in cancers have not been extensively studied. Previous studies showed that LAGE3 was one of the most frequently upregulated RNA modification-related proteins in multiple cancer types [14]. The knockdown of LAGE3 significantly reduces cell proliferation in non-small-cell lung carcinoma cell lines [39]. Here, we aimed to explore the clinical value, potential biological function, and immune-related effects of the LAGE3 protein in CM.

In this study, we profiled the expression of LAGE3 based on TCGA, GEO, HPA databases, and it was upregulated in CM cell lines. Furthermore, survival analyses showed that high LAGE3 expression correlated with a worse prognosis in the CM. However, LAGE3 had no significant correlation with prognosis in patients with primary CM. Coupled with the Cox regression analyses, these data affirm that LAGE3 could be a potential prognostic marker in CM, especially for metastatic patients.

Many cancer-associated and immune-related pathways of LAGE3 were enriched and identified by GO, KEGG, and GSEA. The cancer-associated pathways included hepatocellular carcinoma, chronic myeloid leukemia, colorectal cancer, and renal cell carcinoma. Interestingly, these pathways have also been significantly associated with neurodegenerative diseases such as Alzheimer's disease, Huntington's disease, and Parkinson's disease. Besides, although LAGE3 is not an immune-related gene, GSEA enriched multiple immune functional gene sets.

Immune infiltration in human tumors is a prognostic factor [40]. Lymphocytes often infiltrate primary CM, and the presence of tumor-infiltrating lymphocytes is a significant and independent positive histologic prognostic factor [41]. Previous findings showed that a higher density of CD8^+^ T cells was most associated with survival, while a higher density of CD45^+^ leukocytes, T cells, and B cells was associated with increased survival in patients with metastatic CM [42]. Immunotherapy is an auspicious and practical treatment option for some patients. High levels of CD8^+^ T cells seemed to mediate tumor regression and better response with immunotherapy [43, 44]. Patients with inflammatory tumor infiltrates are thus more likely to benefit from immune checkpoint therapies [45]. Based on the functional enrichment analysis of the LAGE3 protein and the correlation with CM and immune infiltration, we demonstrated that the expression of LAGE3 had a negative correlation with the immune cell infiltration in the CM. Besides, it has been reported that LAGE3 was negatively correlated with the levels of infiltration for multiple immune cells, especially CD8^+^ T cells in colorectal cancer and clear cell renal cell carcinoma [16, 17].

In this study, we used both EPIC and TIMER to analyze immune infiltration. Except for EPIC and quanTIseq, all other *in silico* cell type deconvolution methods provide scores in arbitrary units, which are only meaningful in relation to another sample of the same dataset. Thus, previous studies recommend EPIC for general-purpose deconvolution in immuno-oncology [46]. However, TIMER estimates only six broad immune cell types: B cells, CD4^+^ T cells, CD8^+^ T cells, macrophages, neutrophils, and dendritic cells. This is advantageous because limiting the number of cell types being interrogated to linearly separable cell types could prevent unstable estimation due to statistical collinearity between cell types with very similar gene expression [47, 48]. Among these types of immune cells, CD4^+^ T cells, B cells, CD8^+^ T cells, and macrophages were highly expressed in metastatic CM compared with primary CM as demonstrated by both EPIC and TIMER. Notably, CD8^+^ T cells had the most difference between the LAGE3^low^ and LAGE3^high^ groups in metastatic CM. In addition, previous studies have demonstrated that high infiltration of CD8^+^ T cells is associated with a favorable prognosis in multiple cancers [49, 50]. Similarly, through the TIMER database, we found that patients with high CD8^+^ T cells had a better outcome in metastatic CM. Moreover, the LAGE3^low^ group, with a higher infiltration level of CD8^+^ T cells, had a better outcome in CM. Indeed, LAGE3 protein seemed to suppress immune-related functions. Altogether, the evidence suggested that LAGE3 could be a potential prognostic marker for patients with CM. Besides, the LAGE3 expression was negatively correlated with the infiltration level of key immune cells like CD8^+^ T cells in metastatic CM. Thus, LAGE3 might inform the development of novel CM immunotherapy.

Nevertheless, since we used a small sample size to verify the predictive value of LAGE3 as a biomarker in CM, there is a need for the inclusion of more tumor samples or cohorts for the Cox regression analysis. Besides, our study revealed a markedly negative correlation between LAGE3 and immune infiltration; thus, the potential biological mechanisms of LAGE3 should be systematically investigated using both *in vivo* models and *in vivo* experiments for tumor-immune interaction analysis. In addition, most of the existing TME computational methods are only limited to genomics, which might be insufficient in dissecting the involved biological processes. Thus, multidimensional omics data is required for more accurate quantitative TME contexture in future studies.

## 5. Conclusion

In summary, we have demonstrated that downregulated LAGE3 is associated with longer clinical survival, higher immune infiltration levels, and more active immune-related function in CM patients. This evidence suggested that the LAGE3 protein could lead to the dysregulation of TIICs in tumor sites. Therefore, LAGE3 protein might be an important prognostic biomarker in CM.

## Figures and Tables

**Table 1:** Summary of the datasets.

GEO/dataset ID	Platform	Samples	Patient stage	Survival
TCGA	RNA-seq	471	I-IV	OS
GSE3189	GPL96	70	I-IV	NA
GSE46517	GPL96	121	I-IV	NA
GSE65904	RNA-seq	214	I-IV	DSS
GSE98394	RNA-seq	78	I-III	OS
GSE22153	RNA-seq	57	IV	OS
GSE19234	GPL570	44	III-IV	PRS, OS

OS: overall survival; DSS: disease-specific survival; PRS: postrecurrence survival.

## Data Availability

The datasets supporting the conclusions of this study can be found in The Cancer Genome Atlas (https://portal.gdc.cancer.gov/), Genotype-Tissue Expression dataset (https://www.gtexportal.org/home/), and NCBI Gene Expression Omnibus (GSE3189, GSE98394, GSE46517, GSE19234, and GSE98394).

## Supplementary Materials

Supplementary 1 Supplementary Table 1: univariate Cox regression for overall survival on TCGA melanoma patients.

Supplementary 2 Supplementary Table 2: univariate Cox regression for disease-specific survival on TCGA melanoma patients.

Supplementary 3 Supplementary Table 3: the coexpressed genes of LAGE3 in skin cutaneous melanoma samples.

Supplementary 4 Supplementary Table 4: the Gene Ontology enrichment analysis of LAGE3 relevant signature genes.

Supplementary 5 Supplementary Table 5: the Kyoto Encyclopedia of Genes and Genomes enrichment analysis of LAGE3 relevant signature genes.

Supplementary 6 Supplementary Figure 1: the signaling pathways identified by GSEA. (a) The enriched gene sets in the hallmark collection by the GSEA with high to low LAGE3 expression. Each line represents one particular gene set with unique color, and upregulated gene sets are located above the *x*-axis; by contrast, the downregulated gene sets lay below the *x*-axis. Only gene sets with FDR *q* < 0.05 were considered significant. And only several leading gene sets were displayed in the plot. (b) GSEA identifies numerous KEGG pathways that are highly enriched in LAGE3 expression. (c) Enriched gene sets in the C7 collection, the immunologic gene sets, by samples of high and low LAGE3 expression. Only several leading gene sets are shown in the plot.
