# Supplementary figures and images for: L Antigen Family Member 3 Serves as a Prognostic Biomarker for the Clinical Outcome and Immune Infiltration in Skin Cutaneous Melanoma
Source: Biomed Res Int. 2021 Mar 18;2021:6648182. doi: 10.1155/2021/6648182 (PMC8000545; doi:10.1155/2021/6648182)

**A**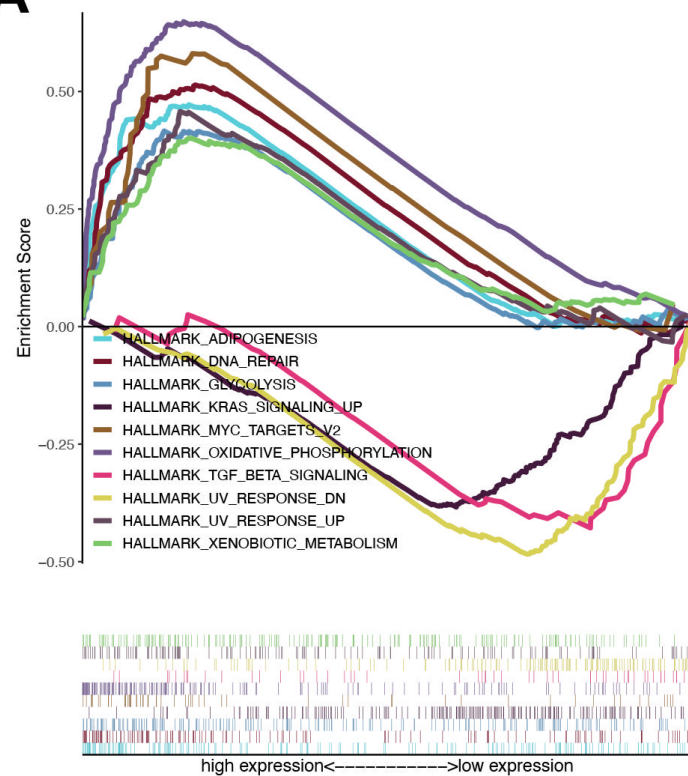**B**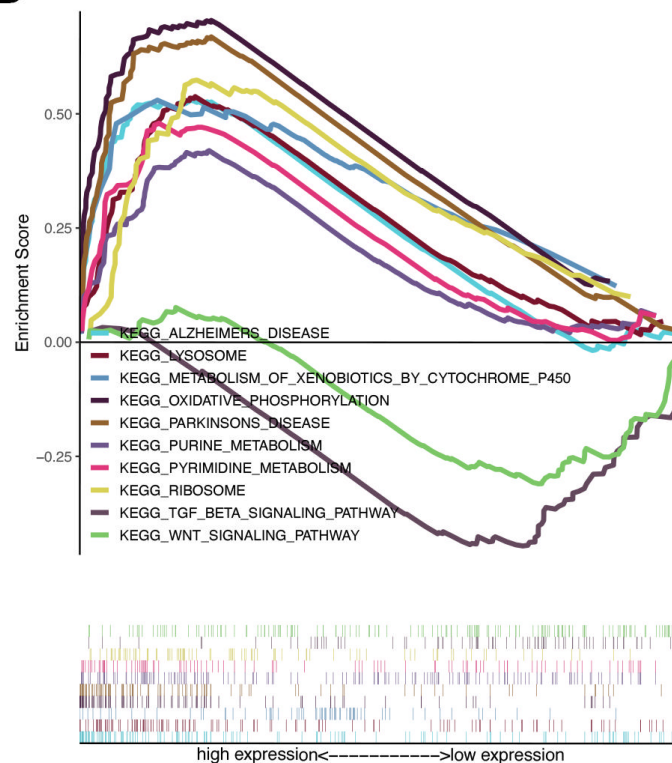**C**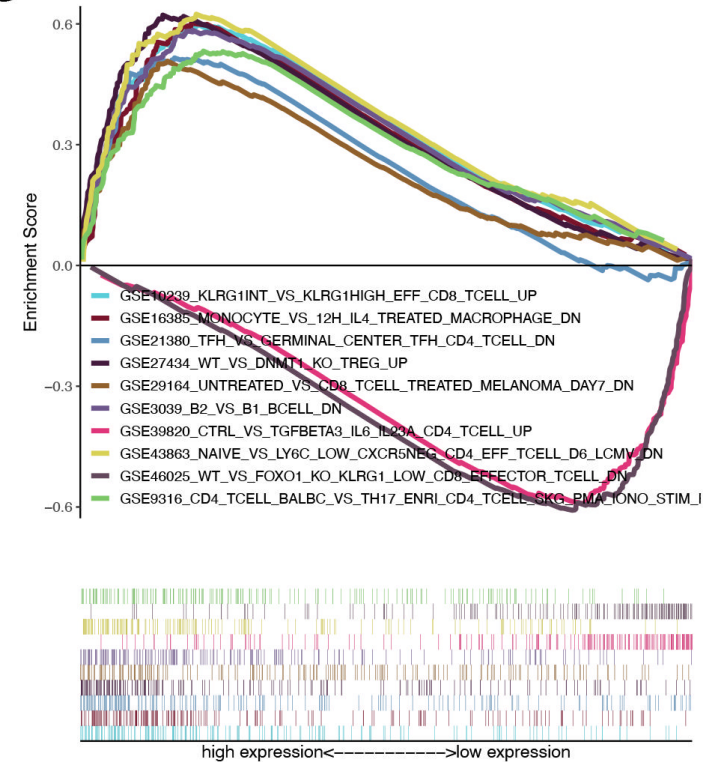

Supplement: Supplementary 6 — Supplementary Figure 1: the signaling pathways identified by GSEA. (a) The enriched gene sets in the hallmark collection by the GSEA with high to low LAGE3 expression. Each line represents one particular gene set with unique color, and upregulated gene sets are located above the x-axis; by contrast, the downregulated gene sets lay below the x-axis. Only gene sets with FDR q < 0.05 were considered significant. And only several leading gene sets were displayed in the plot. (b) GSEA identifies numerous KEGG pathways that are highly enriched in LAGE3 expression. (c) Enriched gene sets in the C7 collection, the immunologic gene sets, by samples of high and low LAGE3 expression. Only several leading gene sets are shown in the plot. [file 6648182.f6.pdf]
